# Agro-based biochars combined with nitrogen fertilizer improve soil nutrient status and rice performance in contrasting soils of southern Nigeria

**DOI:** 10.1038/s41598-026-56968-7

**Published:** 2026-06-18

**Authors:** Adebayo Jonathan Adeyemo, Simon Nwafor Ogboji, Adeyemi Samuel Ayorinde, Evelyn Atinuke Akinnagbe, Adetoyosi Catherine Ariyo, Moses Adeyemi Awodun

**Affiliations:** 1 Department of Crop, Soil and Pest Management, Federal University of Technology, Akure, Nigeria; 2 Department of Crop Production, Federal College of Agriculture, Akure, Nigeria; 3 Department of Crop, Soil and Pest Management, Olusegun Agagu University of Science and Technology, Okitipupa, Nigeria; 4 Department of Agricultural Technology, Federal Polytechnic Ile-Oluji, Ile Oluji, Nigeria; 5 Department of Information Systems and Analytics, University of Arkansas at Little Rock, Little Rock, AR USA

**Keywords:** Agro-wastes, Biochar, Nutrient retention, Rice ecology, Alfisol, Ultisol, Soil fertility, Ecology, Ecology, Environmental sciences, Plant sciences

## Abstract

Agro-based biochars used together with mineral nitrogen fertilizer are increasingly recognized as climate-smart soil amendments for improving soil fertility and sustaining crop productivity. However, field information on the effects of rice husk biochar, sawdust biochar, and urea on post-harvest soil nutrient status across contrasting rice ecosystems in Nigeria remains limited. This study evaluated the effects of rice husk biochar, sawdust biochar, and urea on post-harvest soil nutrient status and rice performance in upland and lowland rice ecosystems established on Alfisol in Akure and Ultisol in Abakaliki, southern Nigeria. The experiment was conducted as a 2 × 2 × 4 factorial in a randomized complete block design with three replicates, involving two soil types, two rice ecologies/varieties, and four nutrient treatments: control, urea, rice husk biochar + urea, and sawdust biochar + urea. Soil pH, soil organic carbon (SOC), total nitrogen (TN), available phosphorus (P), exchangeable potassium (K), ammonium-N (NH_4_^+^-N), nitrate-N (NO_3_^-^-N), and rice yield components were assessed and analysed by factorial ANOVA, with mean separation using Tukey’s HSD at *P* < 0.05. Biochar-urea combinations improved post-harvest soil nutrient status and rice performance relative to the control and, in most cases, sole urea, with the strongest responses occurring in the 0–15 cm soil layer. Sawdust biochar + urea produced the highest grain yield of 4202.59 kg ha^-1^, straw yield of 4999.21 kg ha^-1^, and 1000-grain weight of 30.25 g, while rice husk biochar + urea produced statistically comparable grain and straw yields. These results indicate that locally available agro-based biochars can improve fertilizer effectiveness and post-harvest nutrient status under the conditions of this study; however, longer-term and rate-response studies are required before broad field recommendations are made.

## Introduction

Rice is one of the key contributors to food security in Nigeria; however, average on-farm yields remain well below attainable yields in many production ecologies. Recent assessments of rice systems in sub-Saharan Africa indicate that yield gaps remain large because of low soil fertility, inefficient nutrient use, limited access to inputs, and poor synchrony between nutrient supply and crop demand^1–3^. In Nigeria and similar tropical environments, these constraints are particularly severe in acidic and strongly weathered soils, where low organic matter, low nitrogen availability, phosphorus fixation, and limited cation exchange capacity reduce the efficiency of mineral fertilizer application^4,5^.

Mineral fertilizers will continue to be important in rice intensification; however, their effectiveness in smallholder systems is often constrained by limited nutrient retention capacity, restricted market accessibility, and poor synchronization between fertilizer nutrient release and crop nutrient demand^2,3,6^. Nitrogen is particularly vulnerable to loss through volatilization, nitrification, denitrification, and possible leaching, especially in coarse-textured and low-CEC tropical soils. Therefore, recent nutrient management strategies increasingly emphasize the integration of mineral fertilizers with soil-improving amendments. Biochar can enhance acidic tropical soils by increasing pH, contributing ash-derived base cations, improving cation exchange capacity (CEC), and increasing retention of nutrient ions such as NH_4_^+^, K^+^, and phosphate^4,7,8^. Previous studies have reported that biochar application can improve soil organic carbon, nutrient availability, CEC, and crop performance in degraded tropical soils, particularly when combined with mineral fertilizer rather than applied alone^4,7,8^.

Rice husk biochar is relevant to rice-based agro-ecosystems because rice husk is locally available around rice mills and can be converted into a stable carbon-rich amendment. Sawdust is also widely generated from sawmills and timber-processing activities in southern Nigeria and therefore represents another accessible feedstock for smallholder-oriented soil improvement. The effectiveness of biochar depends on feedstock type, pyrolysis condition, application rate, soil texture, soil acidity, and whether it is applied alone or with fertilizer^7–10^. Studies have shown that biochar combined with mineral fertilizer can improve nutrient retention in the topsoil, increase pH and exchangeable bases, and enhance crop growth more consistently than fertilizer alone or biochar alone^4,6,8,11^. However, these responses are soil-specific and may vary between Alfisols and Ultisols because of differences in acidity, clay content, organic matter, and nutrient-retention capacity.

Nigeria is a significant case study for testing this interaction because rice is cultivated in different ecologies and soil types, including more fertile Alfisols and more weathered Ultisols. Available agro-residues, such as rice husk and sawdust, are significant biochar feedstocks, offering a chance for connecting waste management with soil quality in rice cultivation in Nigeria. However, there is little information on the effect of biochar and urea combinations on post-harvest nutrient distribution across different soil types, ecologies, and soil depth in southern Nigeria. This is significant because the pattern of nutrient retention in the soil might vary in Alfisol and Ultisol systems, as well as in the top and subsoil, with significant implications for fertilizer efficiency and post-harvest soil quality^4,11^.

Against this background, the study assessed the effect of rice husk biochar and sawdust biochar, each combined with urea fertilizer, on post-harvest soil nutrient status and rice performance in contrasting upland and lowland rice ecosystems in southern Nigeria. Specifically, the objectives were to: (i) assess the effect of the treatments on rice grain yield, straw yield, 1000-grain weight, and harvest index; (ii) evaluate treatment effects on post-harvest nutrient status across 0–15 and 15–30 cm soil depths in Alfisol and Ultisol systems; and (iii) quantify the effects of rice husk biochar + urea and sawdust biochar + urea on selected post-harvest soil chemical properties, including pH, SOC, TN, available P, exchangeable K, NH_4_^+^-N, and NO_3_^-^-N.

## Materials and methods

### Study sites and experimental conditions

The field experiment was carried out during the 2019 cropping season at two locations in southern Nigeria representing contrasting soils. Akure in southwestern Nigeria represented an Alfisol, while Abakaliki in southeastern Nigeria represented an Ultisol. Each location included upland and lowland rice production environments in order to represent contrasting rice ecologies. In Akure, the study was carried out at the Federal University of Technology, Akure, while in Abakaliki, it was carried out at Agbalabor Central and North Ameka, Ezza. The contrasting sites allowed comparison of biochar-urea effects under different soil and ecological conditions^11,12^. Weather conditions during the experiment were typical of the humid tropical climate of both locations. Total rainfall during the experimental period was 1,259.84 mm in Akure and 1,376.68 mm in Abakaliki, while the mean temperature was 24.37 degrees C in Akure and 25.76 degrees C in Abakaliki.

### Experimental design and treatments

The experiment was laid out as a 2 × 2 × 4 factorial in a randomized complete block design (RCBD) with three replicates. The factors were soil type/location (Alfisol and Ultisol), rice ecology/variety combination (upland Irat 109 and lowland Upla 3), and nutrient treatment. Because each variety was grown in its corresponding ecology, varietal effects are interpreted as ecology/variety responses rather than as independent genotype effects. The nutrient treatments were: T0, control or no nutrient addition; T1, urea only; T2, rice husk biochar + urea; and T3, sawdust biochar + urea. The biochars were applied at 3 t ha^−1^ and urea at 30 kg N ha^−1^. These rates were selected based on field-manageable rates used in previous rice-biochar and integrated nutrient management studies in tropical soils^6,9,11^. For each location, a main plot measuring 20 m x 15–16 m was divided into subplots measuring 5 m x 5 m with 0.5 m alleys between plots. Rice husk was collected from a local rice mill at Ogbese, Akure, Ondo State, while sawdust was collected from a homogeneous source identified as *Ficus mucuso* (Obobo). The feedstocks were selected because they are locally available agro-industrial residues relevant for smallholder-oriented biochar production. The biochars were produced by oxygen-limited pyrolysis at about 350 degrees C for 55 min using a batch reactor. After cooling, the biochars were applied one month before transplanting and incorporated into the 0–5 cm soil layer to maximize contact with the biologically active topsoil layer.

### Crop establishment and field management

Seeds of Irat 109 and Upla 3 were collected from the International Institute of Tropical Agriculture (IITA), Ibadan, Nigeria. Seed viability before planting was approximately 97% based on pre-sowing assessment. Nursery establishment and transplanting were scheduled to ensure that seedlings of similar physiological age were transplanted across the sites despite ecological differences in moisture conditions. Seedlings were transplanted at 20 cm x 20 cm spacing using four seedlings per hill and later thinned to two seedlings per hill two weeks after transplanting to maintain a uniform stand. This spacing corresponds to an approximate plant population of 250,000 hills ha^-1^ before thinning and a final stand of about 500,000 plants ha^[-1^ after retaining two seedlings per hill. Weeding was carried out manually throughout the season, and plots were protected from vertebrate pests using wire netting. Other crop management practices were kept uniform across treatments so that crop responses could be attributed mainly to biochar and urea application.

### Soil analyses and plant measurements

Growth parameters were recorded at four-week intervals up to 12 weeks after planting (WAP). Parameters recorded included the number of leaves, the number of productive tillers, and plant height. Five representative plants were selected from the central rows of each subplot and repeatedly measured throughout the study period. At harvest, grain yield, straw yield, 1000-grain weight, and harvest index were recorded. Grain yield and straw yield were adjusted to standard moisture content and converted to a hectare basis for treatment comparison^13,14^. After harvest, soil samples were collected from 0 to 15 and 15–30 cm depths in both upland and lowland systems to assess treatment effects on nutrient distribution in the soil profile. The measured soil parameters were pH, soil organic carbon (SOC), total nitrogen (TN), available phosphorus (P), exchangeable potassium (K), ammonium-N (NH_4_^+^-N), and nitrate-N (NO_3_^-^-N), because they are directly relevant to nutrient availability, retention, and fertilizer-use efficiency in acidic tropical rice soils. Soil pH was measured in a 1:2.5 soil: water suspension using the procedure of McLean^15^. Soil organic carbon was determined by the Walkley and Black wet oxidation method^16^, and organic matter was estimated by multiplying SOC by 1.724. Total nitrogen was determined using the Kjeldahl digestion method of Bremner and Mulvaney^17^. Available phosphorus was extracted using the Bray-1 method of Bray and Kurtz^18^, which was selected because the experimental soils were acidic and Bray-1 is appropriate for acidic tropical soils. Exchangeable potassium was extracted with neutral 1 N ammonium acetate and determined using flame photometry following standard procedures. Cation exchange capacity (CEC) was determined by saturating the soil with neutral 1 N ammonium acetate buffered at pH 7.0 and expressing the retained exchangeable cations on a cmol kg-1 basis. Effective cation exchange capacity (ECEC) was calculated as the sum of exchangeable bases and exchangeable acidity. Inorganic nitrogen was extracted with 2 M KCl and determined using procedures consistent with Keeney and Nelson^19^.

### Statistical analysis

Data were analyzed using factorial analysis of variance (ANOVA) to determine the main effects of soil type/location, ecology/variety combination, nutrient treatment, and their interactions. The experimental design was a 2 × 2 × 4 factorial arrangement in a randomized complete block design with three replications. Where significant effects were detected, means were separated using Tukey’s honestly significant difference (HSD) test at *P* < 0.05. Treatment differences described as significant in the text refer only to comparisons supported by the ANOVA and Tukey’s HSD results. Statistical procedures followed standard approaches for factorial field experiments^20,21^.

## Results

### Initial soil and biochar characteristics

The initial soil and biochar characteristics prior to treatment application are presented in Table 1. The soils were acidic, but the Ultisol showed stronger acidity and generally lower fertility than the Alfisol. Across upland and lowland ecologies, the Alfisol generally had higher available P, exchangeable K, and CEC, whereas the Ultisol had higher exchangeable acidity. Nutrient concentrations also varied with depth, with most nutrient values declining in the 15–30 cm layer relative to the 0–15 cm layer. Both rice husk and sawdust biochars were alkaline, with pH values of 8.60 and 8.09, respectively, indicating potential liming effects. Rice husk biochar had a total organic carbon of 50.07% and a CEC of 17.68 cmol kg-1, while sawdust biochar had a higher total organic carbon of 60.59% and a CEC of 27.05 cmol kg^−1^. These properties indicate that both biochars could contribute to nutrient retention, with sawdust biochar showing relatively greater carbon and exchange-capacity characteristics. Available P in the biochars was 680 mg kg⁻¹ for rice husk biochar and 700 mg kg⁻¹ for sawdust biochar. In addition, the CEC determined with neutral 1 N ammonium acetate at pH 7 is theoretically expected to equal or exceed ECEC in these acidic soils because pH-dependent charge is more fully expressed at pH 7. Therefore, the few instances where ECEC slightly exceeded CEC are attributed to analytical and rounding variation between separate determinations rather than to a true reversal of the expected relationship.

**Table 1:** Initial soil chemical properties of the Alfisol and Ultisol at 0–15 and 15–30 cm under upland and lowland ecologies, and selected chemical properties of the rice husk and sawdust biochars used in the study.

VARIABLES	ALFISOL					ULTISOL			BIOCHARS	
	UPLAND LOWLAND UPLAND LOWLAND									
	0–15	15–30	0–15	15–30	0–15	15–30	0–15	15–30	RICE HUSK	SAW DUST
_pH_ (H_2_O)	4.86	4.9	4.61	4.99	4.03	4.09	4.47	4.24	8.6	8.09
Total Organic Carbon (%)	0.39	0.68	0.1	0.16	0.11	0.33	0.58	0.43	50.07	60.59
Total Organic Matter (%)	0.67	1.17	0.17	0.27	1.91	0.57	1	0.19	86.32	ND
Total Nitrogen (g/kg)	0.32	0.31	0.65	0.32	0.1	0.11	0.16	0.1	0.46	0.38
P (mg/kg)	29.78	15.09	40.73	19.86	5.68	2.3	14.89	8.63	680	700
K (cmol/kg)	2.99	3.01	3.98	2.88	0.17	0.16	0.29	0.2	10.3	16.42
Na (cmol/kg)	0.36	0.73	0.58	0.2	0.15	0.19	0.3	0.17	1.96	2
Ca (cmol/kg)	3.16	1.63	0.4	0.63	2.64	2.4	3.2	2.88	4.55	4.62
Mg (cmol/kg)	2.28	1.46	0.18	0.61	1.05	0.34	1.32	1.19	0.87	0.98
CEC (cmol/kg)	8.79	6.83	5.14	3.87	4.01	3.09	5	4.44	17.68	27.05
E. Acidity (cmol/kg)	0.39	0.4	0.43	0.32	2.69	3.15	3.98	3.79	-	-
ECEC (cmol/kg)	9.18	7.23	5.57	4.64	6.7	6.98	8.98	8.23	-	-
Base Saturation (%)	95.75	94.47	92.28	93.1	59.85	49.52	56.18	54.35	-	-
NH_4_-N (ug/g)	0.3	0.29	0.34	0.33	0.27	0.27	0.28	0.31	-	-
NO_3_-N (ug/g)	0.28	0.28	0.3	0.29	0.23	0.25	0.3	0.55	-	-

ND = not determined for biochar organic matter because the conventional SOC × 1.724 conversion is intended for mineral soils and may overestimate organic matter in carbon-rich biochars. CEC was determined using neutral 1 N ammonium acetate buffered at pH 7.0, whereas ECEC was calculated as the sum of exchangeable bases and exchangeable acidity. The slight cases in which ECEC exceeded CEC are attributed to analytical and rounding variation between separate determinations. Base saturation was calculated as (sum of exchangeable bases/ECEC) × 100.

### Crop response associated with improved nutrient status

Rice growth and yield components responded to nutrient treatments in both soils (Table 2). Nutrient treatment significantly affected grain yield, straw yield, and 1000-grain weight, whereas harvest index varied within a narrower and statistically similar range among treatments. Grain yield increased from 1721.24 kg ha^−1^ in the control to 2376.44 kg ha^−1^ under urea, 3940.28 kg ha^−1^ under rice husk biochar + urea, and 4202.59 kg ha^−1^ under sawdust biochar + urea. Straw yield followed a similar pattern, increasing from 2529.01 kg ha-1 in the control to 3479.05, 4725.85, and 4999.21 kg ha^−1^ under urea, rice husk biochar + urea, and sawdust biochar + urea, respectively. The 1000-grain weight increased from 21.34 g in the control to 28.22 g under urea, 30.21 g under rice husk biochar + urea, and 30.25 g under sawdust biochar + urea. Alfisol produced a higher grain yield of 3407.45 kg ha^−1^ and straw yield of 4322.08 kg ha^−1^ than Ultisol, which produced 2962.82 kg ha^−1^ and 3544.48 kg ha^−1^, respectively. The lowland ecology/variety combination (Upla 3) produced a higher grain yield of 3677.30 kg ha^−1^ and a straw yield of 4338.20 kg ha^−1^ than the upland ecology/variety combination (Irat 109), which produced 2692.97 kg ha^−1^ and 3528.36 kg ha^−1^, respectively. These values should be interpreted as combined ecology/variety responses because the two varieties were grown in different rice ecologies.

**Table 2:** Main effects of soil type, variety, nutrient treatment, and their interactions on rice yield components under contrasting soils of southern Nigeria.

FACTORS	Grain Yield	Straw Yield	1000 Grain yield Weight	Harvest Index
	Kg/ha		(g)	
Soil Type (ST) Alfisol (L1) Ultisol (L2) Varieties (V) Irat (109) (V1) Upla (3) (V2) Treatments (T) Check (T0) Urea (T1) RH + Urea (T2) SD + Urea (T3)	3407.45a 2962.82b 2692.97b 3677.30a 1721.24c 2376.44b 3940.28a 4202.59a	4322.08a 3544.48b 3528.36b 4338.20a 2529.01c 3479.05b 4725.85a 4999.21a	28.45a 26.56a 26.85a 28.17a 21.34b 28.22a 30.21a 30.25a	44.60a 45.54a 45.44a 44.71a 46.52a 42.87a 45.33a 45.57a
Model statistics				
ST x V ST x T V x T ST x V x T SD CV	* * * * 12.71 39.91	* * * * 12.72 32.33	* * * * 6.31 22.94	* * * * 6.39 14.17

Means followed by the same letter within the same column are not significantly different according to Tukey’s test at *P* < 0.05. NS = not significant; * = significant at *P* < 0.05. RH + Urea = rice husk biochar + urea (T2); SD + Urea = sawdust biochar + urea (T3).

### Soil organic carbon and total nitrogen distribution

Post-harvest SOC and TN varied with soil type, treatment, depth, and ecology (Tables 3 and 4). In the upland 0–15 cm layer, SOC was highest under rice husk biochar + urea with 2.04%, followed by sawdust biochar + urea with 1.50%, urea with 1.44%, and the control with 1.38%. Total N in the same layer was 0.20, 0.28, 0.35, and 0.27 g kg^−1^ under the control, urea, rice husk biochar + urea, and sawdust biochar + urea, respectively. In the lowland 0–15 cm layer, SOC was also highest under rice husk biochar + urea with 1.38%, while sawdust biochar + urea recorded 0.99%, urea 1.01%, and the control 0.87%. Total N in the lowland 0–15 cm layer was 0.10, 0.16, 0.25, and 0.15 g kg^−1^ under the control, urea, rice husk biochar + urea, and sawdust biochar + urea, respectively. In the 15–30 cm layer, treatment differences were less consistent and generally smaller than in the surface layer. Therefore, the strongest and most consistent responses of SOC and TN occurred in the topsoil, particularly under rice husk biochar + urea, while the subsoil responses were more variable.

**Table 3:** Effects of biochar–urea treatments, soil type, and their interaction on selected post-harvest chemical properties of upland soils at 0–15 and 15–30 cm depths.

FACTORS	(0–15 cm)									(15–30 cm)							
	pH		SOC		TN	*P*	K		pH		SOC	TN		*P*			K
	(1:2.5)		(%)		(g/kg)	(mg/kg)	(cmol/kg)				(%)	(g/kg)		(mg/kg)			(cmol/kg)
	Weeks After Planting																
**Soil Type (ST)**																	
Alfisol (L1)	5.00^a^		1.61^a^		0.30^a^	9.96^b^	0.24^a^		4.91^a^		0.36^a^	0.04^a^		5.50^a^			0.14^a^
Ultisol (L2)	4.03^b^		1.11^b^		0.16^b^	14.06^a^	0.17^b^		4.06^b^		0.34^a^	0.05^a^		4.34^a^			0.16^a^
**Treatments (T)**																	
Check (T0)	4.88^a^		1.38^c^		0.20^b^	4.11^b^	0.09^c^		4.28^a^		0.19^a^	0.02^a^		6.74^a^			0.08^b^
Urea (T1)	4.21^a^		1.44^b^		0.28^a^	4.05^c^	0.14^b^		4.36^a^		0.25^a^	0.04^a^		6.26^a^			0.14^a^
RH + Urea (T2)	4.67^a^		2.04^a^		0.35^a^	10.98^b^	0.26^a^		4.43^a^		0.88^a^	0.04^a^		3.08^a^			0.14^a^
SD + Urea (T3)	4.67^a^		1.50^a^		0.27^a^	21.01^a^	0.21^ab^		4.66^a^		0.48^a^	0.05^a^		5.42^a^			0.16^a^
**Model Statistics** ST T	* NS		* *		* *	* *	* *		* NS		NS NS	NS NS		NS NS			NS *
ST x T	*		*		*	*	*		*		*	*		*			NS
Standard Deviation (SD)	0.68		0.83		0.16	12.55	0.09		0.57		0.19	4.00		0.03			0.05
Coefficient of Variance (CV)	15.07		61.31		69.36	104.47	43.46		12.67		56.25	81.32		62.33			32.86

Means followed by the same letter within the same column are not significantly different according to Tukey’s test at *P* < 0.05. NS = not significant; * = significant at *P* < 0.05. RH + Urea = rice husk biochar + urea (T2); SD + Urea = sawdust biochar + urea (T3).

**Table 4:** Effects of biochar–urea treatments, soil type, and their interaction on selected post-harvest chemical properties of lowland soils at 0–15 and 15–30 cm depths.

FACTORS	(0–15 cm)								(15–30 cm)					
	pH		SOC		TN	*P*	K		pH	SOC	TN	*P*	K	
	(1:2.5)		(%)		(g/kg)	(mg/kg)	(cmol/kg)		(1:2.5)	(%)	(g/kg)	(mg/kg)	(cmol/kg)	
	Weeks After Planting													
Soil Type (ST)														
Alfisol (L1)	5.47a		1.68a		0.29a	7.54a	0.34a		5.55a	0.82a	0.13a	6.66a	0.27a	
Ultisol (L2)	4.47b		0.58b		0.08b	5.91b	0.21b		4.26b	0.42b	0.15a	3.24a	0.16b	
Treatments (T)														
Check (T0)	4.82a		0.87b		0.10a	4.56b	0.16a		4.92a	0.37ab	0.07b	3.47b	0.12b	
Urea (T1)	4.78a		1.01b		0.16a	4.79b	0.21a		4.84a	0.45b	0.22a	4.34ab	0.15b	
RH + Urea (T2)	5.04a		1.38a		0.25a	4.27b	0.30a		5.01a	0.71a	0.11b	3.50b	0.27a	
SD + Urea (T3)	5.10a		0.99b		0.15a	11.12a	0.31a		4.86a	0.70ab	0.10b	7.03a	0.22ab	
Model statistics ST T	* NS		* *		* NS	* *	* NS		* NS	* *	NS *	NS *	* *	
ST x T	NS		*		NS	*	NS		*	*	*	*	*	
Standard Deviation (SD)	0.58		0.80		5.50	0.15	0.15		0.71	0.28	4.41	0.17	0.10	
Coefficient of Variance (CV)	11.64		70.68		81.77	81.62	54.09		14.54	46.64	95.18	123.22	47.51	

Means followed by the same letter within the same column are not significantly different according to Tukey’s test at *P* < 0.05. NS = not significant; * = significant at *P* < 0.05. RH + Urea = rice husk biochar + urea (T2); SD + Urea = sawdust biochar + urea (T3).

### Ammonium-N and nitrate-N distribution

**Fig. 1:**
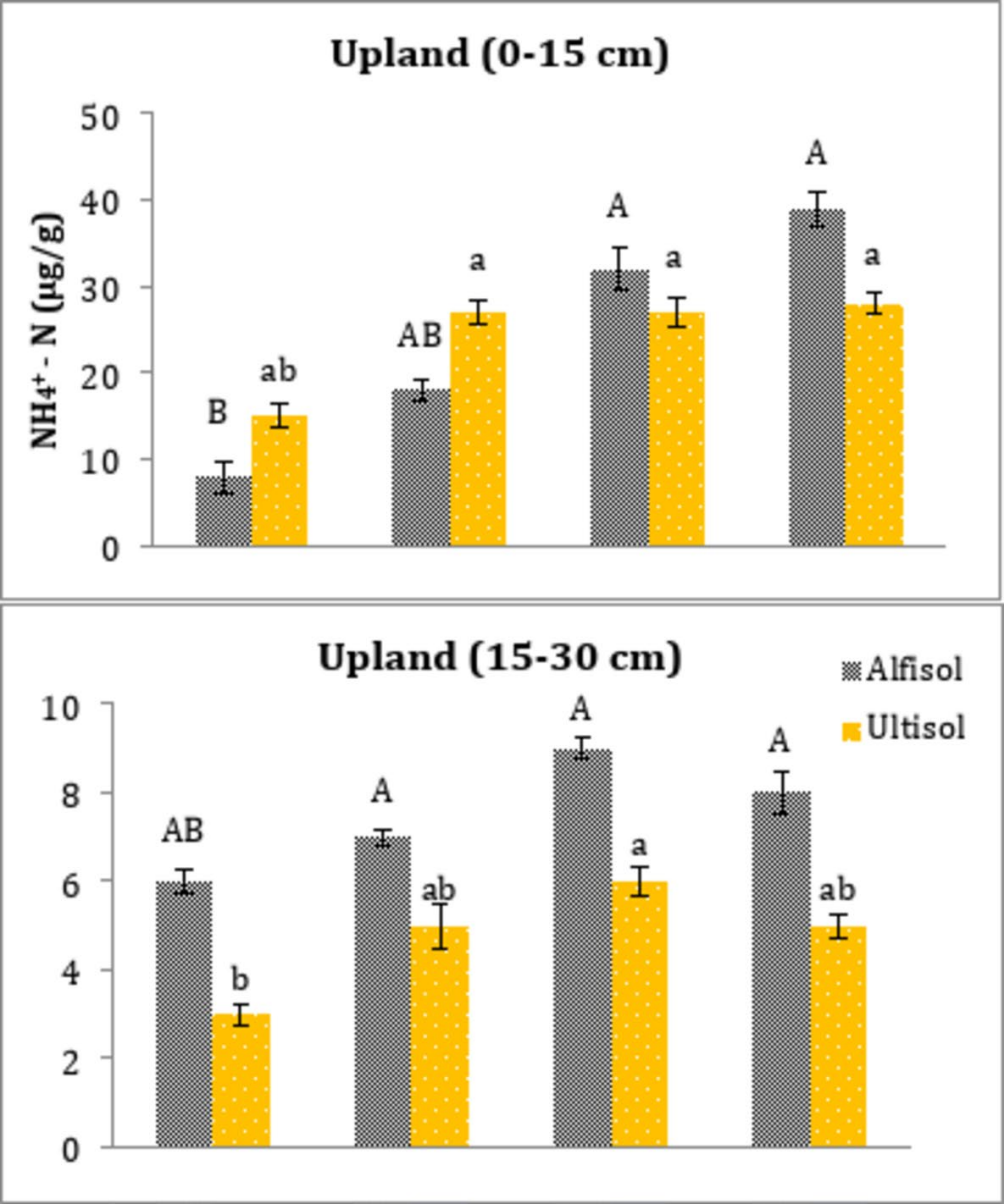

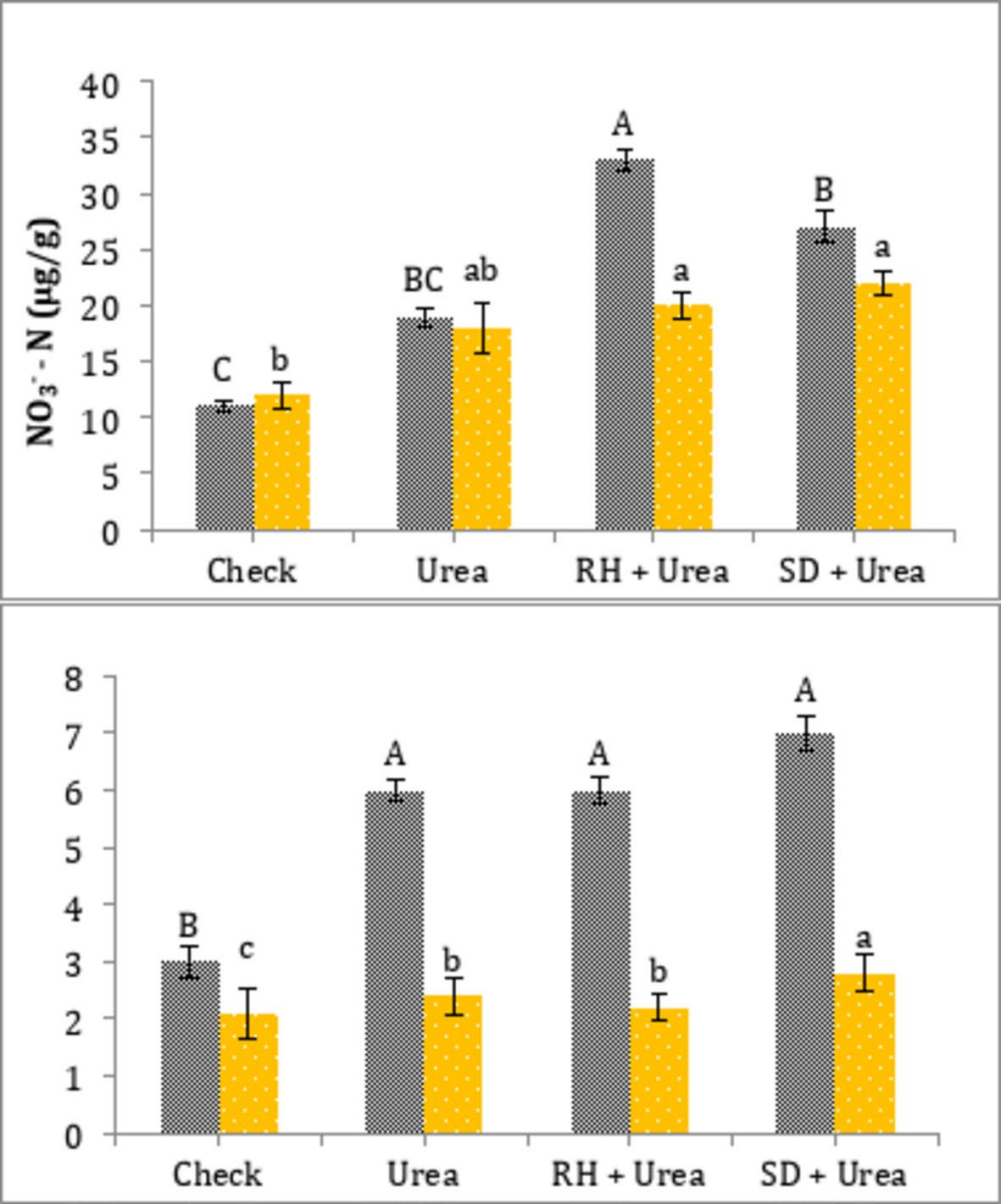
Distribution of ammonium-N (NH_4_^+^-N) and nitrate-N (NO_3_^−^-N) in upland soils at 0–15 and 15–30 cm depths under biochar–urea. treatments in Alfisol and Ultisol of southern Nigeria. Vertical bars represent standard errors of the means. Within each soil depth, bars. bearing the same uppercase letter for Alfisol and the same lowercase letter for Ultisol are not significantly different at *P* < 0.05.

**Fig. 2:**
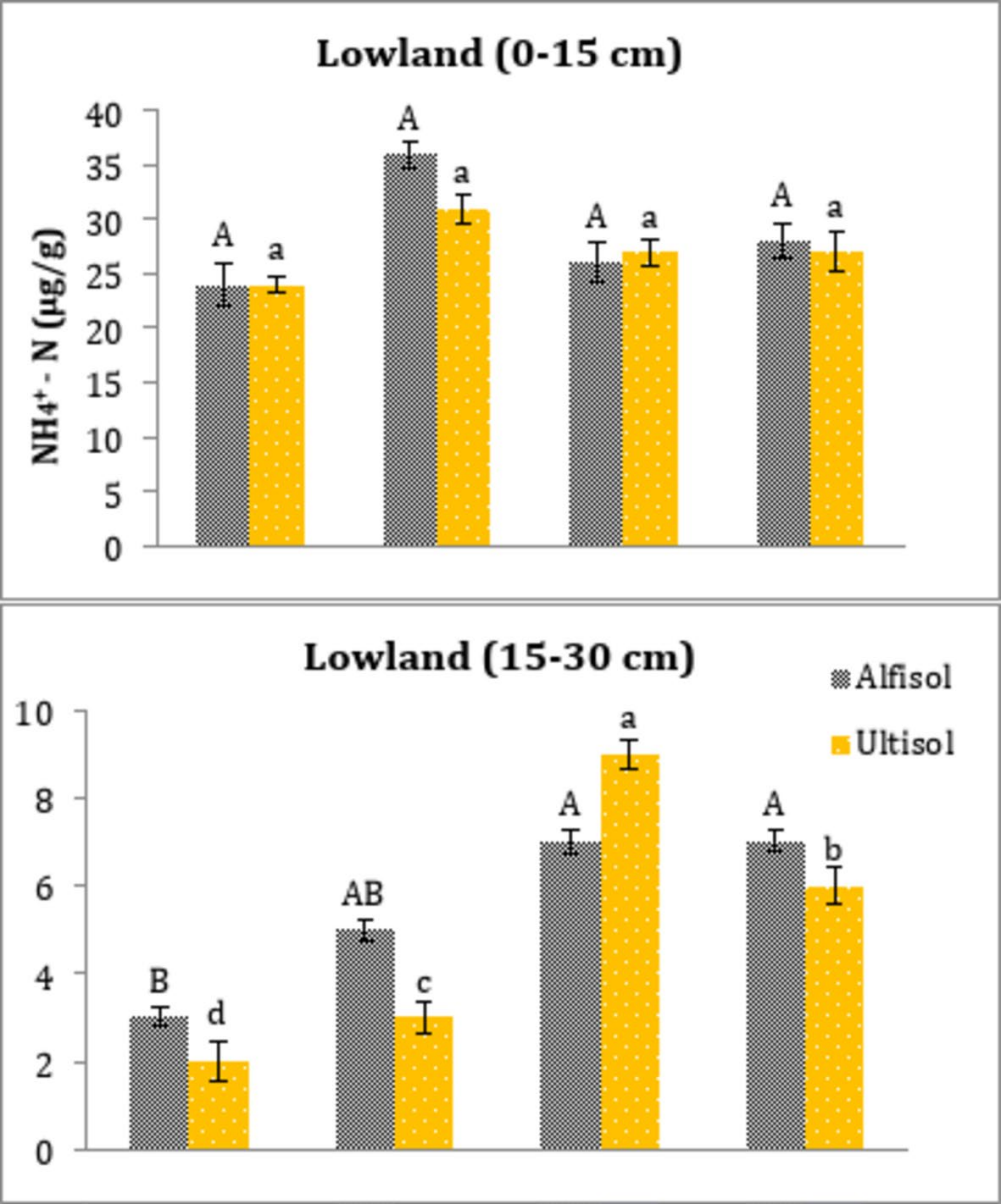

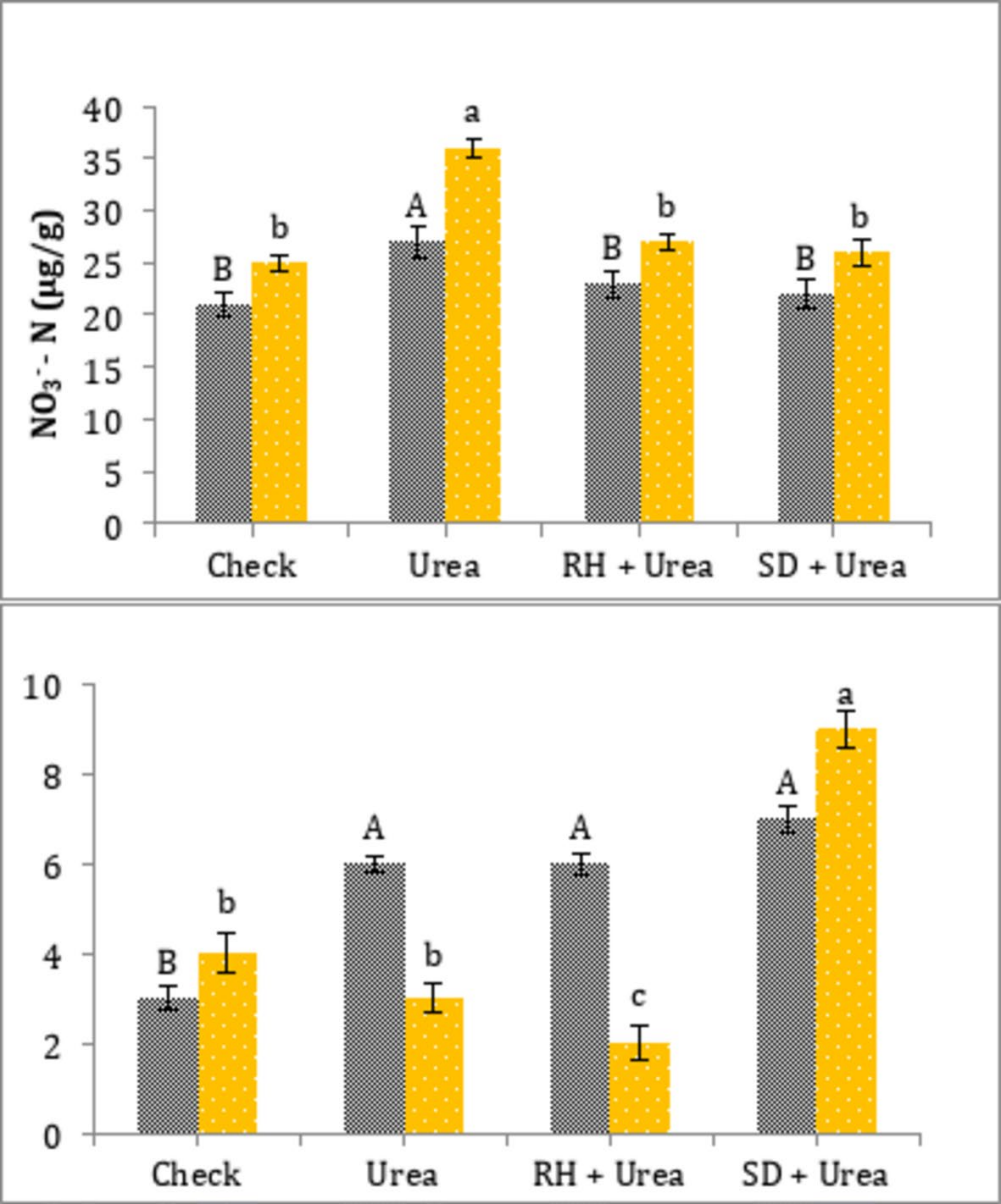
Distribution of ammonium-N (NH_4_^+^-N) and nitrate-N (NO_3_^−^-N) in lowland soils at 0–15 and 15–30 cm depths under biochar–urea. treatments in Alfisol and Ultisol of southern Nigeria. Vertical bars represent standard errors of the means. Within each soil depth, bars bearing. the same uppercase letter for Alfisol and the same lowercase letter for Ultisol are not significantly different at *P* < 0.05.

The distribution of inorganic N forms after harvest varied with soil depth, soil type, and treatment (Figs. 1 and 2). In general, NH_4_^+^-N and NO_3_^-^-N concentrations were higher in the 0–15 cm layer than in the 15–30 cm layer, indicating greater inorganic N concentration in the surface soil where biochar was incorporated. However, because no lysimeter or leachate collection was included, reduced nitrate leaching was not directly measured and is interpreted only as a possible explanation based on soil-profile distribution. For consistency with Figs. 1 and 2, inorganic N was reported as µg g⁻¹ throughout the manuscript. In the upland 15–30 cm soil layer, NH4⁺-N values remained low across treatments, ranging from 0.21 to 0.29 µg g⁻¹, while NO3⁻-N values ranged from 0.21 to 0.28 µg g⁻¹. These small differences indicate that treatment effects on inorganic N in the lower layer should be interpreted cautiously. Overall, the results indicate stronger accumulation of inorganic N in the surface layer than in the lower layer, but they do not provide direct evidence of leaching reduction.

### Soil pH, available phosphorus, and exchangeable potassium

Soil pH, available phosphorus (P), and exchangeable potassium (K) were affected by nutrient treatments, although the magnitude of response varied with soil type, ecology, and soil depth (Tables 3 and 4). In the upland soils, pH was generally higher in the Alfisol than in the Ultisol. However, treatment effects on pH were moderate compared with the responses observed for available P and exchangeable K. In the upland 0–15 cm soil layer, sawdust biochar + urea (T3) recorded the highest available P value of 21.01 mg kg⁻¹, followed by rice husk biochar + urea (T2) with 10.98 mg kg⁻¹. The control and urea-only treatments recorded lower values of 4.11 mg kg⁻¹ and 4.05 mg kg⁻¹, respectively. Exchangeable K was also improved by the biochar–urea treatments, with T2 recording 0.26 cmol kg⁻¹ and T3 recording 0.21 cmol kg⁻¹, compared with 0.09 cmol kg⁻¹ in the control and 0.14 cmol kg⁻¹ in the urea-only treatment. In the lowland 0–15 cm soil layer, T3 also recorded the highest available P value of 11.12 mg kg⁻¹ and the highest exchangeable K value of 0.31 cmol kg⁻¹. Rice husk biochar + urea (T2) also improved exchangeable K to 0.30 cmol kg⁻¹, compared with 0.16 cmol kg⁻¹ in the control and 0.21 cmol kg⁻¹ under urea alone. Overall, the responses were stronger in the 0–15 cm layer than in the 15–30 cm layer, indicating that the biochar–urea effects were more pronounced in the amended surface soil. These results show that biochar combined with urea improved available P and exchangeable K more consistently than urea alone, with sawdust biochar + urea showing the strongest response for available P.

## Discussion

### Baseline soil contrast and implications for amendment response

The contrast in baseline fertility status influenced treatment responses (Table 1). The Alfisol had higher baseline available P, exchangeable K, and CEC, whereas the Ultisol had higher exchangeable acidity. These differences suggest that treatment effects were partly conditioned by soil buffering capacity and initial fertility status. Although poorer soils can sometimes show stronger relative responses to amendments, the Ultisol in this study showed smaller absolute changes for several fertility parameters, possibly because stronger acidity, lower CEC, and lower initial nutrient reserves limited nutrient retention after amendment application^4,8,22^.

### Effects of biochar–urea combinations on soil organic carbon and nitrogen retention

The biochar-urea combinations improved SOC and TN mainly in the 0–15 cm soil layer, where the amendments were incorporated and where root activity and nutrient cycling are usually greatest (Tables 3 and 4). In the upland topsoil, rice husk biochar + urea recorded the highest SOC of 2.04% and TN of 0.35 g kg^−1^, while sawdust biochar + urea also improved SOC and TN relative to the control. In the lowland topsoil, rice husk biochar + urea recorded the highest SOC of 1.38% and TN of 0.25 g kg^−1^. These effects are consistent with the addition of stable carbon through biochar and possible improvement in N retention through increased exchange sites, surface adsorption, and reduced rapid fertilizer-N loss. However, where treatment means were statistically similar, the results are described as trends rather than confirmed significant differences.

### Ammonium and nitrate distribution

The distribution of NH_4_^+^-N and NO_3_^-^-N provides additional evidence on post-harvest inorganic N status, but it should not be interpreted as a direct measurement of leaching (Figs. 1 and 2). The generally higher concentrations of inorganic N in the 0–15 cm layer than in the 15–30 cm layer suggest that more mineral N remained in the amended surface soil after harvest. Mechanistically, NH_4_^+^-N may be retained on negatively charged exchange sites, whereas NO_3_^-^-N remains more mobile in soil solution. Biochar may therefore contribute to fertilizer-N retention by increasing exchange surfaces and improving the chemical environment of the topsoil^22–25,28^. Nevertheless, because leachate was not collected and 15 N tracing was not used, the study can only infer a possible reduction in downward N movement from soil-profile concentrations, not directly confirm leaching reduction.

### Effects on soil pH, available phosphorus, and exchangeable potassium

The responses of soil pH, available P, and exchangeable K demonstrate that biochar-urea combinations influenced post-harvest soil chemical status, especially in the 0–15 cm layer (Tables 3 and 4). In upland topsoil, available P was highest under sawdust biochar + urea of 21.01 mg kg-1, followed by rice husk biochar + urea of 10.98 mg kg^−1^, while the control and urea treatments recorded 4.11 and 4.05 mg kg^−1^, respectively. In the same layer, exchangeable K increased from 0.09 cmol kg^−1^ in the control to 0.14, 0.26, and 0.21 cmol kg^−1^ under urea, rice husk biochar + urea, and sawdust biochar + urea, respectively. In lowland topsoil, sawdust biochar + urea recorded the highest available P of 11.12 mg kg^−1^ and exchangeable K of 0.31 cmol kg^−1^, while rice husk biochar + urea also improved exchangeable K of 0.30 cmol kg^−1^. These improvements may be attributed to the alkaline and nutrient-bearing nature of the biochars, as well as their capacity to increase sorption sites and reduce nutrient loss from the amended surface layer^8,26,27,29–34^.

### Translation of improved nutrient status into rice growth and yield response

The yield results show that improvements in post-harvest nutrient status were associated with better rice productivity (Table 2). Both biochar + urea combinations produced higher grain yield, straw yield, and 1000-grain weight than the control, and they also exceeded urea alone for grain and straw yield. Sawdust biochar + urea produced the highest grain yield of 4202.59 kg ha^−1^, straw yield of 4999.21 kg ha^−1^, and 1000-grain weight of 30.25 g, although grain and straw yields were statistically comparable to rice husk biochar + urea. Harvest index did not differ significantly among nutrient treatments, suggesting that the treatments improved productivity mainly by increasing total biomass and grain yield rather than changing dry-matter partitioning. The stronger yield response under biochar + urea supports the interpretation that biochar improved the effectiveness of urea through better topsoil nutrient status, particularly in relation to SOC, P, and K. However, because the study was conducted for one season and at one biochar rate, these findings should be interpreted as site- and season-specific^35,36^.

### Limitations of the study

This study has some limitations that should be considered when interpreting the findings. First, the experiment was conducted for one cropping season; therefore, the results do not capture long-term biochar ageing, residual effects, or multi-season yield stability. Second, only one biochar rate of 3 t ha^−1^ and one urea rate of 30 kg N ha^−1^ were tested, so rate-response relationships cannot be generalized. Third, nitrate leaching was not directly measured using lysimeters, drainage collection, or isotope tracing; therefore, statements on reduced N movement are based only on post-harvest soil-profile concentrations and should be interpreted cautiously. Fourth, Irat 109 and Upla 3 were grown in their respective upland and lowland ecologies, meaning that variety effects are confounded with ecology and should be interpreted as ecology/variety responses. Finally, the study focused mainly on selected post-harvest soil chemical properties and yield components; additional biological, microbial, and greenhouse-gas measurements would strengthen future mechanistic interpretation.

## Conclusion

The study showed that rice husk biochar + urea and sawdust biochar + urea improved selected post-harvest soil fertility indicators and rice yield components relative to the control under the conditions of the experiment. Treatment effects were strongest in the 0–15 cm soil layer, where biochars were incorporated. Rice husk biochar + urea produced the highest upland topsoil SOC of 2.04% and TN of 0.35 g kg-1, while sawdust biochar + urea produced the highest upland topsoil available P of 21.01 mg kg-1, the highest overall grain yield of 4202.59 kg ha-1, straw yield of 4999.21 kg ha-1, and 1000-grain weight of 30.25 g. Relative to the control, rice husk biochar + urea increased grain yield by about 128.9%, straw yield by about 86.9%, and 1000-grain weight by about 41.6% in the overall treatment means. Relative to the control, sawdust biochar + urea increased grain yield by about 144.2%, straw yield by about 97.7%, and 1000-grain weight by about 41.8% in the overall treatment means. The results indicate that agro-based biochars combined with urea can improve post-harvest nutrient status and rice productivity in acidic tropical soils, but broad recommendations should be made cautiously because the study covered only one season, one biochar rate, and did not directly measure leaching losses.

## Data Availability

The data used for this study will be available on request from the corresponding author.
